# Current practice of pathologic response assessment following chemoimmunotherapy for non‐small cell lung cancer (NSCLC) in Germany: first real‐world data from the multicentre Re‐GraDE study

**DOI:** 10.1111/his.15550

**Published:** 2025-09-05

**Authors:** Felix Elsner, Christiane Kümpers, German Ott, Julia Döring, Katharina Schildknecht, Annette Fisseler‐Eckhoff, Maximilian von Laffert, Lea Baier, Luca Giulini, Dietmar Kraus, Jozef Zustin, Florian Weber, Tamas Szöke, Merle Kögel, Annette Arndt, Verena Büchele, Hanibal Bohnenberger, Florian Fuchs, Christian Matek, Konrad Steinestel, the Pulmonary Pathology Working Group of the German Society of Pathology

**Affiliations:** ^1^ Institute of Pathology University Hospital Erlangen Erlangen Germany; ^2^ Institute of Pathology University Hospital Schleswig‐Holstein Lübeck Germany; ^3^ Department of Clinical Pathology Robert Bosch Hospital and Dr. Margarete Fischer‐Bosch Institute of Clinical Pharmacology Stuttgart Germany; ^4^ Department of Pathology und Cytology Dr. Horst‐Schmidt‐Hospital Wiesbaden Wiesbaden Germany; ^5^ Institute of Pathology University Hospital Leipzig Leipzig Germany; ^6^ Department of Surgery Klinikum Nuernberg, Paracelsus Medical University Nuremberg Nuremberg Germany; ^7^ Gemeinschaftspraxis Für Pathologie Regensburg Regensburg Germany; ^8^ Institute of Pathology University of Regensburg Regensburg Germany; ^9^ Department of Thoracic Surgery Krankenhaus Barmherzige Brüder Regensburg Regensburg Germany; ^10^ Institute of Pathology and Molecular Pathology Bundeswehrkrankenhaus Ulm Ulm Germany; ^11^ Institute of Pathology University Medical Center Göttingen Göttingen Germany; ^12^ Department of Medicine 1 University Hospital Erlangen Erlangen Germany

**Keywords:** major pathologic response (MPR), neoadjuvant immuno‐chemotherapy, NSCLC, pathological complete response (pCR), PD‐L1, residual viable tumour (RVT)

## Abstract

**Background:**

Given that pathologists now frequently assess pathologic response following neoadjuvant or perioperative chemoimmunotherapy for NSCLC, we set up a multicentre study to evaluate the current practice of regression grading in Germany (Re‐GraDE NSCLC).

**Methods:**

133 cases of NSCLC resection specimens following chemoimmunotherapy (IO) were collected from 9 high‐volume lung cancer centres in Germany. Case characteristics were obtained from pathology reports/electronic medical records. In 107 cases, pretreatment biopsies were available on‐site.

**Results:**

Residual viable tumour (% RVT) was commonly used to measure therapy response (106/133 resection specimens, 79.7%). The entire tumour bed was submitted for histology in 55.6% of cases; however, in 18%, a tumour bed of ≤3 cm was not completely submitted. Either Junker or IASLC regression grading was applied in 97.7% of primary tumours and 60.2% of lymph nodes with comparable results. Almost half of the tumours (45.9%) showed pathological complete response (pCR and/or regression grade (RG) III) with a very weak correlation between % RVT and pretreatment PD‐L1 TPS (*r* ^2^ = 0.078, *P* = 0.007). Pretreatment PD‐L1 levels ranged from 0% to 100% (median, 60%) in cases with complete regression, and pCR was observed in 40% of cases with pretreatment PD‐L1 TPS <1%.

**Conclusions:**

Our multicentre study describes the current practice of histopathological regression grading of NSCLC after IO in Germany, highlighting the widespread use of the Junker system, which is basically comparable to IASLC regression grading. For standardization, we recommend following the IASLC guidelines (submitting of the complete tumour bed if ≤3 cm), while the reporting of % RVT might represent a continuous parameter for therapy response. Our digital nationwide registry, which aims to integrate biopsy results, molecular profiling and % RVT in resection specimens, might develop into a valuable tool to investigate novel predictive biomarkers of IO efficacy.

## Graphical abstract

Re‐GraDE, a multicentre German study including 133 cases, highlights variability in regression grading, macroscopic tumour workup, and use of Junker/IASLC systems after neoadjuvant immuno‐chemotherapy in NSCLC. Results from Junker and IASLC systems are comparable. A weak correlation was found between pretreatment PD‐L1 status and residual viable tumour percentage.

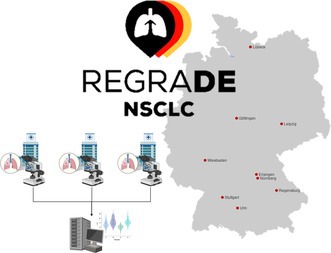

**Abbreviations**
CPS: combined positivity score
IC: immune cell score
IO: immune‐oncology
LN: lymph node
MPR: major pathologic response
NSCLC: non‐small cell lung cancer
pCR: pathological complete response
PD‐L1: programmed death ligand 1
PT: primary tumour
RG: regression grade
RVT: residual viable tumour
TPS: tumour proportion score

## Introduction

Immune checkpoint blockade (ICB) therapies, such as anti‐PD‐1/PD‐L1 agents, enhance antitumoral immune response and have shown impressive improvement of outcomes in the adjuvant setting in non‐small cell lung cancer (NSCLC).^1^, ^2^ In the neoadjuvant or perioperative setting, five recent trials (NEOTORCH, Checkmate 816, Checkmate 77T, AEGEAN and Keynote 671) demonstrated that the addition of immunotherapy to neoadjuvant chemotherapy improved event‐free survival along with significantly higher rates of pathological complete response (pCR, 0% residual viable tumour, RVT) and major pathologic response (MPR, ≤10% RVT) in the surgical resection specimen.^3^, ^4^, ^5^, ^6^, ^7^ In addition, improvement of overall survival could be shown in some of these studies (e.g. Keynote 671). Thorough reviews on the state of pathologic response assessment of lung resection specimens after neoadjuvant therapy have recently been provided by Dacic and by Berezowska *et al*.^8^, ^9^ While the first standardized protocols for pathologic response assessment of lung resection specimens after (radio)chemotherapy date back to 1997,^10^ these lack detailed recommendations for standardized macroscopic assessment (including lymph nodes) and propose a 4‐tiered regression grading (I, IIA, IIB and III) rather than reporting % RVT as a continuous variable.^11^ In 2020, the International Association for the Study of Lung Cancer (IASLC) published guidelines on how to process and evaluate resected lung cancer specimens after neoadjuvant therapy for clinical trials and clinical practice.^12^ It is not stated in full detail in the above‐mentioned trials, which led to the approval of neoadjuvant or perioperative chemoimmunotherapy, if and to what extent the IASLC recommendations, including macroscopic assessment of the resection specimen, were followed.

Given that pathologists are now frequently facing the task to assess pathologic response following chemoimmunotherapy for NSCLC, in 2024, the thoracic pathology working group of the German Society of Pathology (DGP) recommended to follow the 2020 IASLC guidelines for the workup of these cases. In detail, the tumour bed (≤3 cm) and lymph nodes (≤2 cm) should be entirely submitted for microscopic evaluation, and % RVT should be reported as a continuous variable. At the same time, we set up a multicentre study for the evaluation of the current practice of regression grading in Germany (Re‐GraDE) to evaluate which protocols are currently employed, to identify possible obstacles in the practice of regression grading, and to set up a large real‐world cohort of NSCLC cases following neoadjuvant therapy to identify predictive variables for therapy response in ongoing studies.

## Cases and Methods

### Case Selection

A total of 133 consecutive cases of NSCLC resection specimens between 11/2019 and 04/2025 were reported from 9 high‐volume lung cancer centres in Germany (Ulm, Erlangen, Lübeck, Wiesbaden, Stuttgart, Göttingen, Leipzig, Nuernberg and Regensburg). The clinicopathological data are summarized in Table 1. Case characteristics were obtained from pathology reports and electronic medical records, collected and evaluated in a central database. Since it was the goal of the study to collect real‐world data on the practice of regression grading in Germany, study centres reported the results of their individual standard operating procedures (SOP). Regarding pretherapeutic biopsies, these were classified as adenocarcinoma/squamous cell carcinoma and NSCLC, NOS following the 2021 WHO classification.^13^ Regular participation in external ring trials by all centres reassured quality and validity of PD‐L1 immunohistochemistry and scoring.^14^ Molecular genetic testing was done by next generation sequencing (NGS) in most cases (83.5%), with isolated *EGFR*/*ALK* testing in 6% and no molecular testing in 10.5% of cases. All anatomic resection specimens included oncologic lymph node dissection as recommended by the ESTS.^15^

**Table 1:** Clinicopathological characteristics of the complete patient cohort and of cases with (ypT0) and without complete regression (ypT1‐4)

		ypT0 (RG III, pCR)	ypT1‐4 (RG I‐II)	*P*‐value
Parameter	*n* = 133 (%)	*n* = 61 (%)	*n* = 72 (%)	
Gender				
Male	70 (52.6)	33 (54.1)	37 (51.4)	0.755
Female	63 (47.4)	28 (45.9)	35 (48.6)	
Age (years)				
≤66	72 (54.1)	26 (42.6)	46 (63.9)	**0.014**
>66	61 (45.9)	35 (57.4)	26 (36.1)	
cT category				
cT1	23 (17.3)	12 (19.7)	11 (15.3)	0.761^a^
cT2	34 (25.6)	15 (24.5)	19 (26.4)	
cT3	26 (19.5)	12 (19.7)	14 (19.4)	
cT4	39 (29.3)	17 (27.9)	22 (30.6)	
cTx	11 (8.3)	5 (8.2)	6 (8.3)	
cN category				
cN0	23 (17.3)	8 (13.1)	15 (20.8)	0.310^b^
cN1	27 (20.3)	15 (24.6)	12 (16.6)	
cN2	67 (50.4)	29 (47.5)	38 (52.8)	
cN3	5 (3.8)	2 (3.3)	3 (4.2)	
cNx	11 (8.3)	7 (11.5)	4 (5.6)	
cM category				
cM0	98 (73.7)	50 (82.0)	48 (66.7)	0.205^c^
cM1	10 (7.5)	3 (4.9)	7 (9.7)	
cMx	25 (18.8)	8 (13.1)	17 (23.6)	
IO therapy				
Pembrolizumab	62 (46.6)	28 (45.9)	34 (47.2)	0.733^d^
Nivolumab	58 (43.6)	28 (45.9)	30 (41.7)	
Atezolizumab	3 (2.3)	2 (3.3)	1 (1.3)	
Cemiplimab	2 (1.5)	0	2 (2.8)	
Durvalumab	3 (2.3)	1 (1.6)	2 (2.8)	
Unknown	5 (3.8)	2 (3.3)	3 (4.2)	
Conventional therapy				
Carbo/Pacli	70 (52.6)	36 (59.1)	34 (47.2)	0.665^e^
Carbo/Pem	28 (21.1)	13 (21.3)	15 (20.8)	
Cis/Vino	6 (4.5)	2 (3.3)	4 (5.6)	
Cis/Pacli	3 (2.3)	2 (3.3)	1 (1.3)	
Cis/Pem	9 (6.8)	4 (6.5)	5 (7.0)	
Other/unknown	17 (12.8)	4 (6.5)	13 (18.1)	
Therapy cycles				
≤3	82 (61.7)	36 (59.1)	46 (63.9)	0.519^f^
>3	42 (31.6)	21 (34.4)	21 (29.1)	
Unknown	9 (6.7)	4 (6.5)	5 (7.0)	
Histology				
Adenocarcinoma	65 (48.9)	27 (44.2)	38 (52.8)	0.294^g^
Squamous cell carcinoma	63 (47.3)	32 (52.5)	31 (43.0)	
Others/NOS/unknown	5 (3.8)	2 (3.3)	3 (4.2)	
PD‐L1 TPS in pretherapeutic biopsy				
August	15 (11.3)	6 (9.8)	9 (12.5)	0.595^h^
≥1%	95 (71.4)	45 (73.8)	50 (69.4)	
No data	23 (17.3)	10 (16.4)	13 (18.1)	
Size of tumour bed				
≤3	52 (39.1)	33 (54.1)	19 (26.4)	**0.007** ^i^
3	47 (35.3)	17 (27.9)	30 (41.7)	
No data	34 (25.6)	11 (18.0)	23 (31.9)	
Regression according to Junker				
I	15 (11.3)	0	15 (20.8)	**<0.001** ^j^
IIB	32 (24.0)	0	32 (44.4)	
IIA	20 (15.0)	0	20 (27.8)	
III	63 (47.4)	61 (100.0)	2 (2.8)	
Not reported	3 (2.3)	0	3 (4.2)	
Regression according to IASLC				
No pCR/MPR	46 (34.5)	0	46 (63.9)	**<0.001** ^k^
MPR	21 (15.8)	0	21 (29.1)	
pCR	63 (47.4)	61 (100.0)	2 (2.8)	
Not reported	3 (2.3)	0	3 (4.2)	
Quantification of residual viable tumour (in %)				
Reported	106 (79.7)	51 (83.6)	55 (76.4)	0.302
Not reported	27 (20.3)	10 (16.4)	17 (23.6)	
Embedding of PT				
Complete	74 (55.6)	44 (72.1)	30 (41.7)	**<0.001** ^l^
Incomplete	47 (35.3)	12 (19.7)	35 (48.6)	
No data	12 (9.1)	5 (8.2)	7 (9.7)	
ypN category				
ypN0	91 (68.4)	56 (91.8)	35 (48.6)	**<0.001** ^m^
ypN1	15 (11.3)	2 (3.3)	13 (18.1)	
ypN2	27 (20.3)	3 (4.9)	24 (33.3)	
ypN3	0	0	0	
Regression according to Junker for LN				
Yes	80 (60.2)	40 (65.6)	40 (55.6)	0.240
No	53 (39.8)	21 (34.4)	32 (44.4)	
Regression according to IASLC for LN				
Yes	80 (60.2)	40 (65.6)	40 (55.6)	0.240
No	53 (39.8)	21 (34.4)	32 (44.4)	

All statistic tests in the table were done for ypT0 (RGIII, pCR) versus ypT1‐4 (RG I/II, no pCR). The values in bold are signifcant (*p* < 0.05).

^a^ Chi^2^ was tested for cT1/2 versus cT3/4 (*n* = 122).

^b^ Chi^2^ was tested for cN0 versus cN1‐3 (*n* = 122).

^c^ Chi^2^ was tested for cM0 versus cM1 (*n* = 108).

^d^ Chi^2^ was tested for pembrolizumab versus nivolumab (*n* = 120).

^e^ Chi^2^ was tested for the regimen containing Carboplatin versus Cisplatin (*n* = 116).

^f^ Chi^2^ was tested for ≤3 cycles versus >3 cycles (*n* = 124).

^g^ Chi^2^ was tested for ADC versus SCC (*n* = 128).

^h^ Chi^2^ was tested for PDL1‐TPS <1% versus ≥1% (*n* = 110).

^i^ Chi^2^ was tested for ≤3.0 cm versus >3.0 cm (*n* = 99).

^j^ Chi^2^ was tested for Junker Grade I‐IIb versus III (*n* = 130).

^k^ Chi^2^ was tested for no MPR/cPR and MPR versus cPR (*n* = 130).

^l^ Chi^2^ was tested for complete versus incomplete embedding.

^m^ Chi^2^ was tested for ypN0 versus ypN1‐3 (*n* = 132).

### Protocols for Regression Grading

Two different protocols have been used to grade regression. The Junker system [10] is a three‐tiered system: Grade I is defined as no or only minor tumour regression. Grade II is characterized by morphological signs of therapy‐induced tumour regression, with Grade IIA containing more than or equal to 10% RVT and Grade IIB less than 10% RVT. Grade III is applied for cases with complete tumour regression (no evidence of viable tumour cells in the primary tumour bed and lymph nodes). In contrast, the IASLC recommendations represent an expert consensus on regression grading in NSCLC resection specimens after any form of neoadjuvant therapy^12^: MPR is characterized by less than or equal to 10% RVT while pCR requires no viable tumour at all. Of note, while MPR corresponds to Junker regression grade IIB in cases with <10% RVT, the definition is different in cases with exactly 10% RVT (MPR, but Junker IIA). Regarding lymph nodes, nodes ≤2 cm were completely embedded and lymph nodes ≥0.5 cm in largest diameter were bivalved. For (rare) lymph nodes exceeding 2 cm in size, at least one representative slice of the largest diameter was embedded. With regard to lymph nodes (LNs), the original Junker system does not apply a separate regression grade to lymph nodes, but rather includes the presence or absence of residual vital tumour in LNs into Grade Groups IIb or III, respectively.^10^ In contrast, the IASLC recommendations explicitly state that the same regression grading approach applied to the primary tumour (PT) can be used for lymph nodes, stating the presence or absence of residual tumour and reporting percent viable tumour, necrosis and stroma.^12^

### Ethics statement

While study protocols covering the Re‐GraDE approach were specifically approved by the ethics committees of the Universities of Ulm (No. 293/24), Lübeck (2025‐161), Erlangen (24‐175‐Bn) and Göttingen (#24‐4‐20), participation of the remaining centres were reported to the local ethics committees following the new “one study, one vote” approach of the Working Group of Medical Ethics Committees (AKEK) in Germany.^16^ The first data presented here represents a secondary analysis of pooled preexisting pathology reports from 9 centres from which no individual patient‐specific characteristics can be drawn.

### Image Creation

Figure 2A,B were in part created in https://BioRender.com.

### Statistical Methods

Statistical analyses were performed using GraphPad Prism 10 (GraphPad Software Inc., Boston, MA, USA) and SPSS software version 28 (SPSS Inc., Chicago, IL, USA). The threshold for statistical significance was *P* < 0.05. Differences between continuous variables were tested by t‐test/ANOVA, while possible correlations were analysed by linear regression. The association between pCR and different clinicopathological features was assessed by Chi^2^‐test. Data analysis for visualization was performed using R (R core team) v4.3.3. Heatmaps were constructed using the ComplexHeatmap package (Gu *et al*., 2016, v2.18.0). Sankey plots were plotted using the ggsankey package (Sjoberg 2025, v.0.0.99999).

## Results

### Neoadjuvant Checkpoint Inhibition and Clinico‐Pathological Parameters

**Figure 1:**
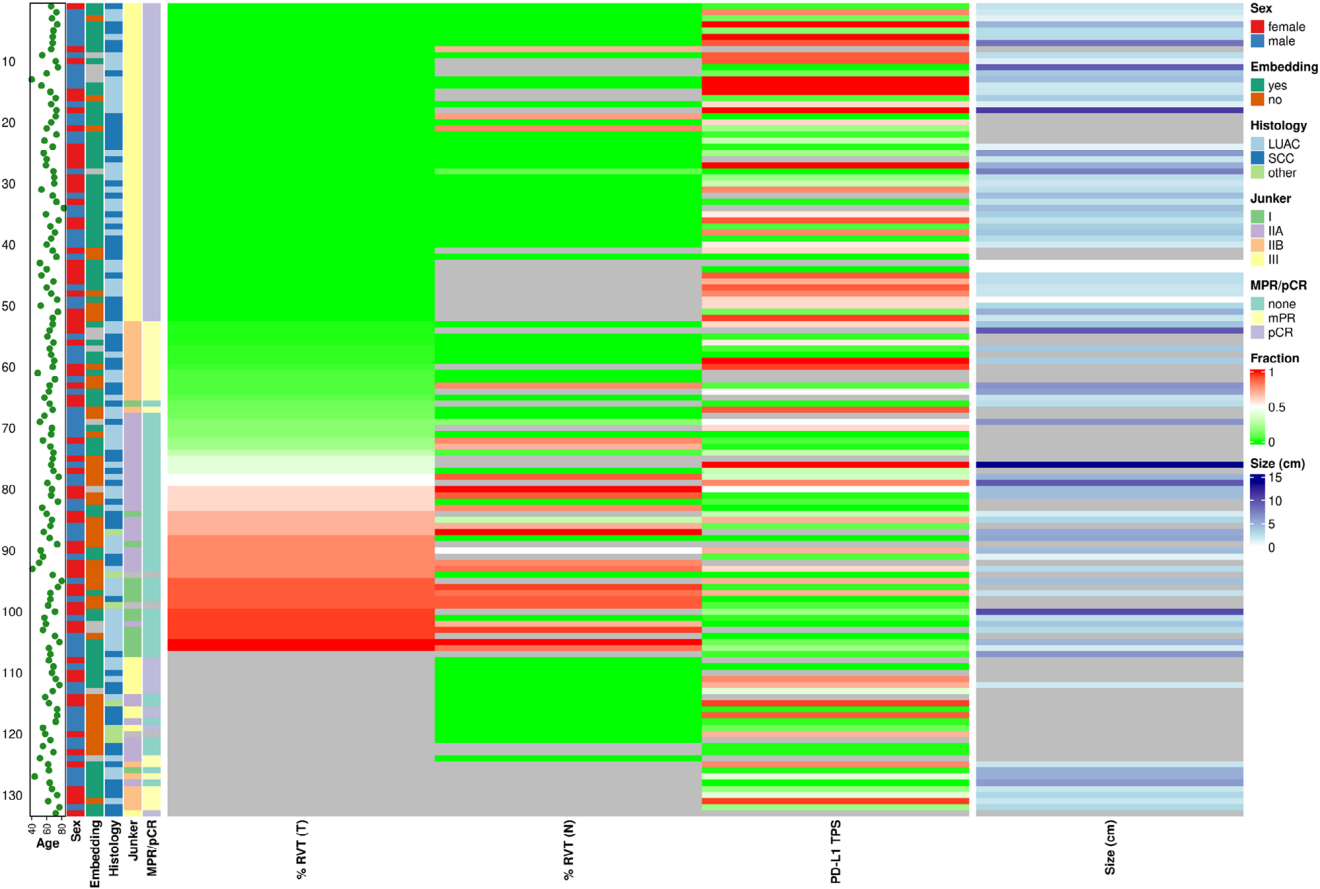
Heatmap summarizing clinic‐pathologic case characteristics and results from regression grading according to Junker/IASLC.

In total, 133 resection specimens from 133 patients (median age, 66; range, 40–83; 70 male, 63 female) after neoadjuvant or perioperative IO therapy were collected. The most frequent IO drug was pembrolizumab (46.6%), followed by nivolumab (43.6%), atezolizumab (2.3%), durvalumab (2.3%) and cemiplimab (1.5%) (Figure 1, Table 1). About two thirds of patients received three or fewer therapy cycles, one third received more than 3 cycles of IO therapy. No significant correlation between administration of any IO therapy and clinic‐pathologic parameters or tumour regression was observed. In most cases, the conventional chemotherapy backbone that paralleled IO treatment contained Carboplatin (73.7%), followed by Cisplatin (13.6%).

### Histology, Immunohistochemistry and Molecular Pathology of Pretreatment Biopsies

**Figure 2:**
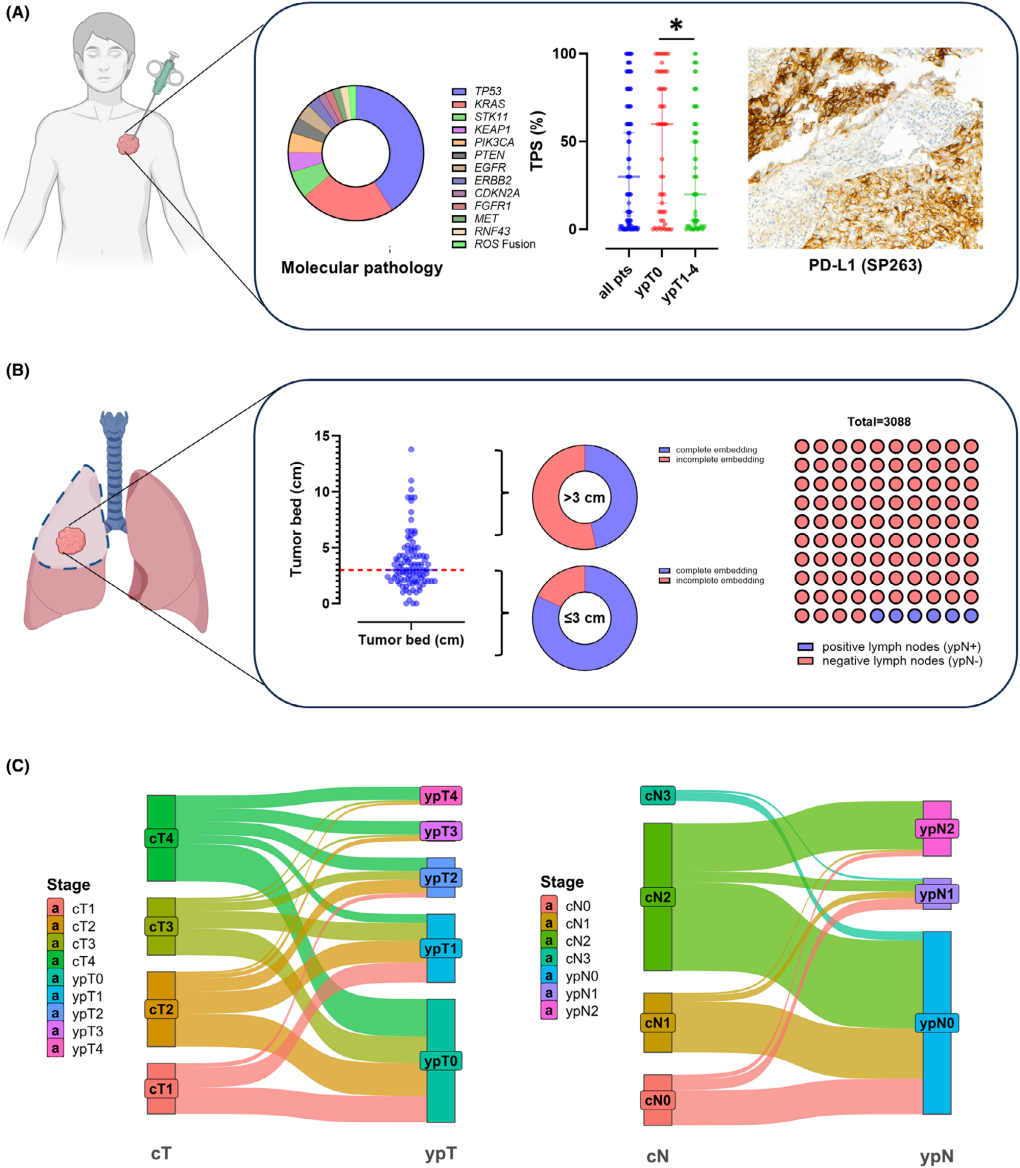
(**A**) Molecular characteristics and results of PD‐L1 TP scoring of pretreatment biopsies. (**B**) Macroscopic workup and lymph node staging of lung resection specimens after neoadjuvant IO treatment. (**C**) cT/ypT and cN/ypN downstaging after neoadjuvant IO treatment.

Out of 133 cases, pretreatment biopsies from 107 cases were diagnosed on‐site in the respective study centre (80.5%). For the remaining 26 patients, information on pretreatment biopsies was gathered from external pathology reports. In 65 out of 133 patients (48.9%), a diagnosis of lung adenocarcinoma was made in the initial biopsy, while 63 biopsies (47.3%) were classified as squamous cell carcinoma. A diagnosis of non‐small cell carcinoma (NOS) was reported in 5 cases (3.8%). Although not recommended in the biopsy setting, histopathological grading was reported in 95 cases (71.4%), with most cases classified as poorly differentiated/high‐grade pattern (G3, 60%). Pretreatment molecular testing (on biopsy) was performed for 119 patients (89.5%) (Figure 2A). Among cases with identifiable driver mutations, the most frequent genomic alterations for all histologic subtypes were point mutations in *TP53* (66.2%) and *KRAS* (36.9%), followed by *STK11* (10.8%), *KEAP1* (7.7%), *PIK3CA* (7.7%), *PTEN* (6.2) and *EGFR* (6.2%) in addition to a variety of less common genomic alterations. All detected genetic alterations are shown in Table S1. The pCR rate in mutation‐specific subgroups ranged from 0% to 100%, with some mutations harboured in only one case. There was information on pretreatment PD‐L1 expression (tumour proportion score, TPS) from 112 patients (84.2%) with a median TPS of 30% (range 0%–100%).

### Workup of Resection Specimens and Lymph Nodes after Neoadjuvant IO Therapy

Macroscopic workup of lung resection specimens after IO therapy was performed by site‐specific SOP and retrospectively evaluated (Figure 2B). Complete embedding was done in 55.6% and incomplete embedding in 35.3% of cases, while in 8.1% no retrospective assessment of the macroscopic workup was possible. Among cases with macroscopic identifiable tumour bed of 3 cm or less, the rate of incomplete submission for histologic evaluation was 18%. On average, 23.2 LN per patient were removed (range 2–67); the average number of LN metastases for node‐positive patients was 4.4 (range 1–28). In total, 3088 lymph nodes were evaluated, of which 193 nodes showed remaining viable tumour (6.3%). There was a significant downstaging under neoadjuvant IO therapy from pretreatment cT/cN stages to posttreatment ypT/ypN stages (Figure 2C).

### Microscopic Assessment of RVT after Neoadjuvant IO Therapy

**Figure 3:**
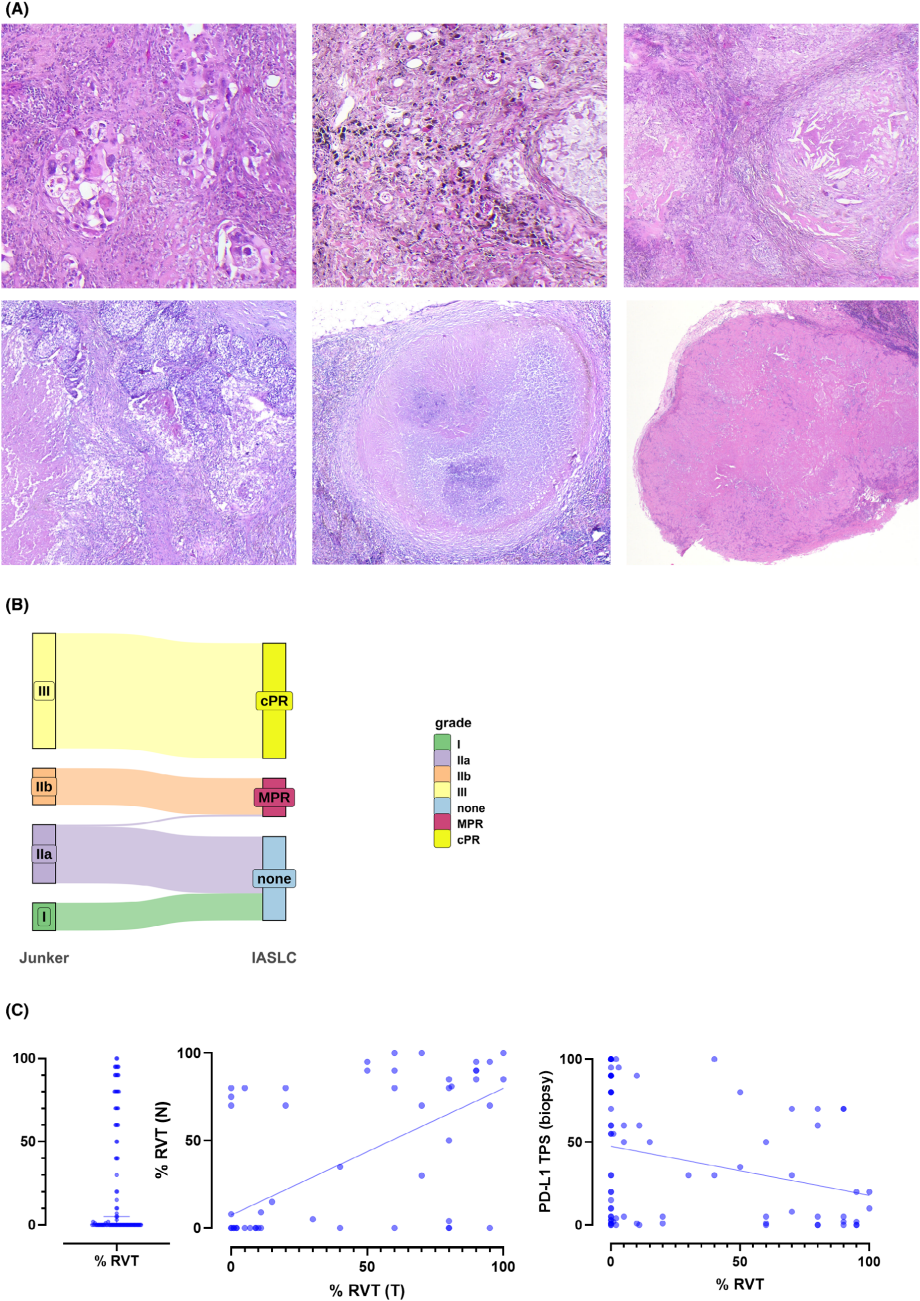
(**A**) representative microphotographs of cases of a primary lung adenocarcinoma (upper panel) and lymph node metastasis of squamous cell carcinoma (lower panel) with residual viable tumour (left) and pathological complete response (centre and right). (**B**) comparable results from Junkerr and IASLC regression grading with only one case (exactly 10% RVT) classified as Junker IIA, but MPR. (**C**) Distribution of % RVT in the investigated specimens, correlation between % RVT in primary tumour and lymph nodes (*r* ^2^ = 0.515452, *P* < 0.0001, linear regression) and between % RVT and pretreatment (biopsy) PD‐L1 TPS (*r* ^2^ = 0.07811, *P* = 0.452007, linear regression). [Colour figure can be viewed at wileyonlinelibrary.com]

Junker/IASLC regression grading was applied in each 97.7% of primary tumours (PT) and 60.2% of lymph nodes (LN) (Figure 3A). Of note, no separate regression grading was reported for LN specimens in 39.8%. All in all, complete regression of the primary tumour was reported in both 63 RG III/pCR cases out of 130 Junker/IASLC‐graded cases (47.4%). There was a high agreement in Junker and IASLC grading, with only one case (of exactly 10% RVT) being graded as Junker IIA, but MPR (Figure 3B). RVT as a continuous variable was assessed in 106 of 133 cases (79.7%), with a median RVT of 1 (range 0%–100%) (Figure 3C). In general, response to IO therapy (as measured by % RVT in the resection specimen) correlated well between PT and LN (*r* ^2^ = 0.452, *P* < 0.0001, linear regression). Nevertheless, we observed all possible scenarios including complete response in both PT and LN, complete response in PT with RVT in LN, RVT in PT with complete response in LN, and RVT in both PT and LN.

### Limited Predictive Value of Pretreatment PD‐L1 Scores

We observed a weak correlation between pretreatment PD‐L1 TPS and %RVT (*r* ^2^ = 0.078, *P* = 0.007, linear regression). Cases with complete regression (0% RVT, RGIII/pCR) had significantly higher pretreatment PD‐L1 TPS (median, 60; range, 0–100) compared to cases with incomplete regression (≥1% RVT; median, 20; range, 0–100; *P* = 0.039; Student's t‐test; Figure 2A). Six out of 15 patients with PD‐L1 TPS of <1/0% in the pretreatment biopsy showed complete regression (0% RVT/RGIII/pCR; 40.0%).

## Discussion

In the light of recent approvals for neoadjuvant and perioperative chemoimmunotherapy in NSCLC, the aim of the Re‐GraDE study was to evaluate the current state of pathologic response assessment following these therapies in Germany. Therefore, we analysed data from 133 resection specimens after IO treatment from 9 high‐volume lung cancer centres, resulting in – to our knowledge – the largest real‐world cohort of NSCLC resection specimens after neoadjuvant treatment so far outside of IO therapy trials. Of note, corresponding pretreatment biopsies were evaluable on‐site in 80.5% of cases compared to 33% in the post hoc analysis of the CheckMate 816 trial.^17^ Most patients were male and had undergone therapy with pembrolizumab and platinum doublet. This contrasts with an earlier evaluation of the data in 12/2024 when nivolumab had been the most frequent IO component, possibly reflecting a shift from neoadjuvant to perioperative treatment. Another contrast to the respective phase 3 trial cohorts is the comparable frequency of adenocarcinoma and squamous histology in our study cohort. Although not currently recommended, histopathological grading was frequently done on biopsy specimens (71.4%), but the reported majority of high‐grade patterns (60%) may point towards a possible overcalling in this setting. The distribution of molecular tumour characteristics (*KRAS, BRAF, TP53, STK11* and *KEAP1* mutations) in the study cohort correlated with the known frequency of mutations, with actionable *EGFR* and *ALK* alterations being under‐represented.^18^, ^19^ This is based on the fact that targeted therapies are available for these patients (although not in the neoadjuvant setting [14–16]), and that *ALK*‐/*EGFR*‐altered tumours have been shown to have a poor response to IO therapy, leading to their exclusion from the CheckMate 816 trial [5, 17]. Still, three patients received IO therapy despite the presence of EGFR mutation. This contrasts with the current guidelines and the individual clinical decisions could not be fully retraced during our retrospective data analysis. One possible scenario could be that IO therapy has been started due to high PD‐L1 expression in all three cases (TPS 60%–90%) and before molecular testing was initiated. We refrained from further analysis of tumour regression among the respective molecular subgroups due to the small sample size. It has also to be noted that comprehensive molecular testing was not performed in all cases, some of which were diagnosed outside of the Re‐GraDE study centres. This heterogeneity reflects the current situation in Germany, where the approach to molecular testing might depend on the setting (academic vs. private), available methods (single‐gene testing vs. NGS) and reimbursement issues.^20^

Both Junker and IASLC regression grading provided robust metrics for pathologic response assessment in terms of RVT. In one case with exactly 10% RVT, there was a discrepancy due to the different definitions of 10% RVT according to the Junker (RG IIA) and IASLC (MPR) grading systems. Despite comparable results, it has to be noted that the use of different grading systems carries a risk of confusing surgeons, oncologists and patients, especially if the extent of macroscopic workup is not clearly defined and if the grading system differs from that used in the relevant pivotal trials. Despite the overall correlation of response to neoadjuvant IO therapy, we observed some heterogeneity in treatment response between PT and LN metastases, which is concordant with existing literature.^21^, ^22^ This finding might reflect different tumour microenvironment and/or genetic or epigenetic heterogeneity between PT and LN metastases.^23^ Reporting a separate regression grading for LN might therefore be beneficial to evaluate the prognostic impact of (different quantities of) residual viable tumour in the LN and for more precise prognostic stratification in cases with heterogeneous response in PT and LN. Overall, neoadjuvant IO therapy resulted in a marked response rate with complete regression of the PT in a substantial proportion of patients. The fact that the observed pCR/RGIII fraction in the investigated real‐world cohort was higher compared to the respective approval studies raises the question of potential under‐sampling in our study.^17^ However, since there is a higher fraction of completely submitted cases in the pCR/RGIII fraction, we conclude that the real‐world efficacy of neoadjuvant/perioperative IO treatment might in fact outperform the results from approval studies.

Regarding the predictive value of PD‐L1 expression in the pretreatment biopsy, the most important finding here is that immunohistochemical PD‐L1 expression in pretreatment biopsies did only weakly correlate with pathologic response to neoadjuvant IO treatment, and more than half of PD‐L1‐negative cases (TPS <1%) showed complete regression. However, it should also be noted here that different PD‐L1 clones were used between the centres, which may limit the general validity of this observation.

Clear limitations of our study lie in the retrospective nature, with patients showing various histologic and molecular profiles without standardized criteria for data assimilation, immunohistochemical and molecular testing. While extensive sampling of the tumour bed (up to complete embedding) was performed in most cases, macroscopic workup could not be assessed in retrospect for 9.1% of patients. As representative sampling up to embedding of the complete tumour bed is crucial for accurate assessment of pathologic response,^8^, ^24^ this represents a possible bias in a subset of patients in our cohort. Furthermore, the small number of patients with specific molecular alterations (e.g. *STK11*/*KEAP1*) precludes a meaningful statistical evaluation of the predictive value of these markers for neoadjuvant IO therapy. Moreover, there is so far no correlation of pathologic response with recurrence and survival data due to the short follow‐up since approval of neoadjuvant IO therapy. Since we also have not yet assessed the interobserver reproducibility of the reported Junker/IASLC gradings, the second step of the nationwide Re‐GraDE study will consist of digitalization and secondary evaluation of all resection specimens and corresponding pretreatment biopsies by multiple investigators, in part assisted by AI‐enhanced technologies.

In conclusion, although both grading systems provided comparable results, there is an unmet need to harmonize protocols for histopathological regression grading of NSCLC resection specimens after neoadjuvant IO treatment. These should include macroscopic evaluation according to the IASLC criteria, representative sampling (up to embedding of the complete tumour bed) and reporting % RVT for both PT and LN as a continuous variable in addition to the Junker and IASLC grading systems. Our nationwide registry, which aims to integrate digitized biopsies and resection specimens as well as molecular and immunohistochemical profiling, will be a valuable tool for investigating novel biomarkers of IO response, which is urgently needed to improve outcomes for NSCLC patients at all stages of the disease.

## Author contributions

FE, CK, VB, CM and KS conceived and planned the study and contributed pathology reports. GO, JD, KS, AFE, MvL, LB, LG, DK, MK, AA, JZ, FW, TS, FF and HB contributed pathology reports and contributed to the interpretation of the results. FE, CM and KS took the lead in writing the manuscript. All authors provided critical feedback and helped shape the research, analysis and manuscript.

## Funding information

There are no current external funding sources for this project.

## Conflict of interests

K Steinestel has received consulting fees, payments or honoraria from: AstraZeneca, Merck Sharp & Dohme, Bristol‐Meyers Squibb, Sanofi and Boehringer Ingelheim. Participation on a Data Safety Monitoring Board or Advisory Board for Merck Sharp & Dohme, Bristol‐Myers Squibb and AstraZeneca. M. von Laffert has received consulting fees, payments or honoraria from: AstraZeneca, Merck, Roche, Sanofi, Boehringer Ingelheim, Janssen, Bristol‐Myers Squibb. C. Kümpers has received consulting fees, payments or honoraria from: AstraZeneca, Merck Sharp & Dohme and Boehringer Ingelheim. All other authors declare no conflict of interest relevant to this study.

## Supporting information


**Table S1.** Overview of all detected genomic alterations in 65 patients with identifiable molecular alterations in the tested regions. Another 54 patients were wild‐type in all analysed genes and not included in this table.

## Data Availability

All data (including access to digital slide scans) is available from the authors upon reasonable request.
