# Supplementary material for: Current practice of pathologic response assessment following chemoimmunotherapy for non‐small cell lung cancer (NSCLC) in Germany: first real‐world data from the multicentre Re‐GraDE study
Source: Histopathology. 2025 Sep 5;87(6):869–79. doi: 10.1111/his.15550 (PMC12605775; doi:10.1111/his.15550)
Supplement: Supplementary file 1 — Table S1. Overview of all detected genomic alterations in 65 patients with identifiable molecular alterations in the tested regions. Another 54 patients were wild‐type in all analysed genes and not included in this table. [file HIS-87-869-s001.docx]

**Supplementary Table 1: Overview of all detected genomic alterations in 65 patients with identifiable molecular alterations in the tested regions. Another 54 patients were wildtype in all analysed genes and not included in this table.**

| **Genomic alteration** | **Frequency n=65 (%)** | **n and % of patients with alteration and pathologic complete response (RGIII/pCR)** |
| --- | --- | --- |
| *TP53* Mutation | 43 (66.2) | 24 (55.8) |
| *KRAS* Mutation | 24 (36.9) | 11 (45.8) |
| *STK11* Mutation | 7 (10.8) | 3 (42.9) |
| *KEAP1* Mutation | 5 (7.7) | 2 (40.0) |
| *PIK3CA* | 5 (7.7) | 3 (60.0) |
| *PTEN* Mutation | 4 (6.2) | 3 (75.0) |
| *EGFR* Mutation | 4 (6.2) | 1 (25.0) |
| *ERBB2* Mutation | 3 (4.6) | 1 (33.3) |
| *CDKN2A* Mutation | 2 (3.0) | 0 |
| *FGFR1* Mutation | 2 (3.0) | 1 (50.0) |
| *MET* Mutation | 2 (3.0) | 2 (100.0) |
| *RNF43* Mutation | 2 (3.0) | 1 (50.0) |
| *ROS Fusion* | 2 (3.0) | 1 (50.0) |
| *ALOX12B* Mutation | 1 (1.5) | 0 |
| *ARID1A* Mutation | 1 (1.5) | 1 (100.0) |
| *BARD1* | 1 (1.5) | 1 (100.0) |
| *BRAF* Mutation | 1 (1.5) | 0 |
| *DNMT3A* | 1 (1.5) | 0 |
| *MAP2K1* Mutation | 1 (1.5) | 0 |
| *MCL1* | 1 (1.5) | 0 |
| *MET low-level amplification (GCN 3.5)* | 1 (1.5) | 0 |
| *MSH2* | 1 (1.5) | 0 |
| *NF1* Mutation | 1 (1.5) | 1 (100.0) |
| *NFE2L2/NRF2* Mutation | 1 (1.5) | 0 |
| *NTRK3* Mutation | 1 (1.5) | 0 |
| *NTRK1* Mutation | 1 (1.5) | 0 |
| *PTCH* | 1 (1.5) | 0 |
| *PTPN11* Mutation | 1 (1.5) | 1 (100.0) |
| *RET* | 1 (1.5) | 1 (100.0) |
| *SMAD4* | 1 (1.5) | 0 |
| *TGFBR2* | 1 (1.5) | 0 |
| *Tyros 3* | 1 (1.5) | 0 |
